# Evaluation of Electronic Cigarette Use (Vaping) Topography and Estimation of Liquid Consumption: Implications for Research Protocol Standards Definition and for Public Health Authorities’ Regulation

**DOI:** 10.3390/ijerph10062500

**Published:** 2013-06-18

**Authors:** Konstantinos E. Farsalinos, Giorgio Romagna, Dimitris Tsiapras, Stamatis Kyrzopoulos, Vassilis Voudris

**Affiliations:** 1 Onassis Cardiac Surgery Center, Sygrou 356, Kallithea 17674, Greece; E-Mails: dtsiapras@hotmail.com (D.T.); stkyrz@gmail.com (S.K.); vvoudris@otenet.gr (V.V.); 2 Abich s.r.l., Biological and Chemical Toxicology Research Laboratory, Via 42 Martiri, 213/B-28924 Verbania (VB), Italy; E-Mail: giorgio.romagna@gmail.com

**Keywords:** electronic cigarette, smoking, vaping, cigarettes, tobacco, topography, nicotine

## Abstract

*Background*: Although millions of people are using electronic cigarettes (ECs) and research on this topic has intensified in recent years, the pattern of EC use has not been systematically studied. Additionally, no comparative measure of exposure and nicotine delivery between EC and tobacco cigarette or nicotine replacement therapy (NRTs) has been established. This is important, especially in the context of the proposal for a new Tobacco Product Directive issued by the European Commission. *Methods*: A second generation EC device, consisting of a higher capacity battery and tank atomiser design compared to smaller cigarette-like batteries and cartomizers, and a 9 mg/mL nicotine-concentration liquid were used in this study. Eighty subjects were recruited; 45 experienced EC users and 35 smokers. EC users were video-recorded when using the device (ECIG group), while smokers were recorded when smoking (SM-S group) and when using the EC (SM-E group) in a randomized cross-over design. Puff, inhalation and exhalation duration were measured. Additionally, the amount of EC liquid consumed by experienced EC users was measured at 5 min (similar to the time needed to smoke one tobacco cigarette) and at 20 min (similar to the time needed for a nicotine inhaler to deliver 4 mg nicotine). *Results*: Puff duration was significantly higher in ECIG (4.2 ± 0.7 s) compared to SM-S (2.1 ± 0.4 s) and SM-E (2.3 ± 0.5 s), while inhalation time was lower (1.3 ± 0.4, 2.1 ± 0.4 and 2.1 ± 0.4 respectively). No difference was observed in exhalation duration. EC users took 13 puffs and consumed 62 ± 16 mg liquid in 5 min; they took 43 puffs and consumed 219 ± 56 mg liquid in 20 min. Nicotine delivery was estimated at 0.46 ± 0.12 mg after 5 min and 1.63 ± 0.41 mg after 20 min of use. Therefore, 20.8 mg/mL and 23.8 mg/mL nicotine-containing liquids would deliver 1 mg of nicotine in 5 min and 4 mg nicotine in 20 min, respectively. Since the ISO method significantly underestimates nicotine delivery by tobacco cigarettes, it seems that liquids with even higher than 24 mg/mL nicotine concentration would be comparable to one tobacco cigarette. *Conclusions*: EC use topography is significantly different compared to smoking. Four-second puffs with 20–30 s interpuff interval should be used when assessing EC effects in laboratory experiments, provided that the equipment used does not get overheated. Based on the characteristics of the device used in this study, a 20 mg/mL nicotine concentration liquid would be needed in order to deliver nicotine at amounts similar to the maximum allowable content of one tobacco cigarette (as measured by the ISO 3308 method). The results of this study do not support the statement of the European Commission Tobacco Product Directive that liquids with nicotine concentration of 4 mg/mL are comparable to NRTs in the amount of nicotine delivered to the user.

## 1. Introduction

Cigarette smoking is a major risk factor for a variety of diseases. Despite the availability of approved medications and aids for smoking cessation, long-term quit rates are relatively low [1]. Therefore, tobacco harm reduction strategy and products have been developed, with the main goal to reduce the amount of harmful substances administered to the human body [2].

Electronic cigarettes (ECs) have been introduced to the market in recent years as an alternative-to-smoking habit. They are battery-driven devices that vaporise a liquid containing mainly nicotine, propylene glycol, glycerine, water and flavourings (according to manufacturers’ reports). By using this device (commonly called “vaping”), nicotine is delivered to the upper and lower respiratory tract without any combustion involved. 

Since ECs are used as substitute for smoking, most studies are oriented towards comparing their effects with those of tobacco cigarettes. Concerning laboratory studies, no consensus has been developed on the way ECs should be handled in the experimental setting and how vapour should be produced in order to test the devices and liquids in conditions simulating real use. The characteristics, mode of function and use patterns of the EC precludes from use the standards applied to tobacco cigarette research [3], such as the ISO 3308 method [4] (35 mL puffs, 2 s per puff, 60 s interpuff interval), for EC testing. Thus, evaluating vaping topography is very important in this aspect. Clinical studies have produced conflicting results on the nicotine-delivery potential of the EC; it seems that there is a learning curve and that experienced users use the device more intensively compared to novice users [5]. In most such studies, smokers who use the EC for the first time are recruited [6,7], and minimal nicotine absorption from EC use was detected. Contrary to these, a small study by Vansickel *et al.* [8] found that nicotine levels were significantly elevated in experienced EC users who were asked to use their own devices. To the best of our knowledge, no study has specifically examined the possible differences in the way ECs are used by experienced compared to novice users. 

Recently the European Commission has issued a proposal for a new Tobacco Product Directive (TPD) [9]. According to this, all nicotine-containing products which either have nicotine level of more than 2 mg, a nicotine concentration exceeding 4 mg/mL or whose intended use results in a mean peak plasma concentration exceeding 4 ng/mL should be authorized as medicinal products before being placed on the market. This decision was established by considering the nicotine content and delivery of medicinal products (nicotine replacement therapies—NRTs) [9]. ECs fall into the category of nicotine-containing products; however, they are marketed as an alternative-to-smoking habit and not as smoking-cessation devices, and their mode of use is different compared to NRTs. Moreover, typical steady-state plasma nicotine concentrations observed from NRTs use range from 10 to 20 ng/mL [10,11]. Finally, 1 mL of liquid as a unit for measuring consumption is not comparable to one cigarette or one pharmaceutical nicotine replacement unit (gum or inhaler cartridge); every puff from an EC leads to vaporisation of small amount of liquid and experienced users have an average daily consumption of 3–4 mL [12]. In any case, no comparative measure of nicotine delivery has been developed between ECs and NRTs or tobacco cigarettes and no study has ever evaluated the amount of EC liquid consumed by experienced users at specific time-intervals of use. Therefore, the purpose of this study was to: (a) examine the patterns of use of EC in experienced and novice users, compared with the use-pattern of tobacco cigarettes, and (b) to assess the liquid consumption and calculate nicotine delivery when ECs are used by experienced users.

## 2. Experimental Section

### 2.1. Study Sample

The study sample consisted of 45 experienced EC users (more than two months of EC use) and 35 smokers (more than one year smoking duration), aged 20–45 years. All EC users were former smokers, by completely substituting smoking with EC. They were using the EC daily, and were consuming liquid with a nicotine concentration of 6–12 mg/mL. Smokers reported that they had never used any type of EC device before participating to this study. Subjects were recruited as part of another protocol evaluating the acute clinical effects of using a “medium-strength” nicotine-containing EC liquid. They were healthy, asymptomatic, did not receive any medications and had normal physical examination and resting electrocardiogram and echocardiogram. They presented for the study after abstaining from food, coffee, alcohol and smoking and EC use for at least eight hours. Informed consent was signed by all subjects before participating to the study. The protocol was approved by the ethics committee of our institution and conforms to the provisions of the Declaration of Helsinki.

### 2.2. Evaluation of Use Pattern

**Figure 1:**
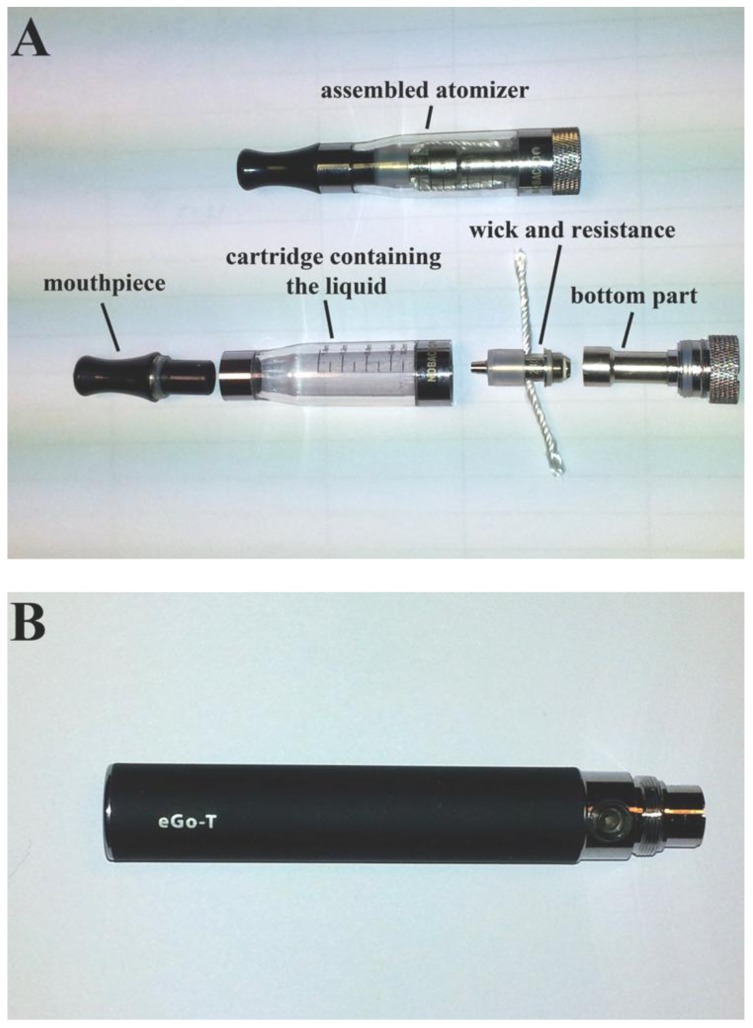
The electronic cigarette device used in the study, consisting of (**A**) the atomizer and its various parts and (**B**) the battery.

Experienced EC users were provided with a fully-charged, manually-activated EC device (“eGo-T” battery with “Epsilon” atomizer, Nobacco—Figure 1). The equipment used is considered “second generation”; the battery has higher capacity compared to cigarette-like devices and the atomizer design is different compared to polyfil-containing cartomizers. A 9 mg/mL nicotine-containing liquid was used, which is considered “medium-strength”. Originally our intention was to test another atomiser (“eGo-C”, Joyetech); however, some EC users experienced overheating of the atomizer and a phenomenon known as “dry-puff” (unpleasant, burning taste caused by insufficient supply of liquid to the resistance so that evaporation rate is higher than liquid supply-see the Discussion section for more details). They had to lower the puff duration and increase the inter-puff interval in order to avoid this phenomenon. In response to that, the eGo-C was substituted with “Epsilon” and participants were asked to come back for recordings with the new atomizer. All recordings with the eGo-C atomizer were discarded.

EC users were asked to use the device *ad lib* for 20 min (ECIG group). Recording was performed by a researcher using a digital camera, taking care to have a clear view of the mouth and of the LED of the activating button of the EC device. Smokers were assigned to smoke cigarettes (SM-S group) and use the EC (SM-E group) on two separate days in a randomized cross-over design. During the smoking session, they were asked to smoke two cigarettes of the same brand, containing 7 mg tar and 0.7 mg nicotine (according to box information), and were recorded by a researcher. Care was taken to obtain a clear view of the mouth during the recording, in order to know when the smoker started inhaling and exhaling smoke. During the EC-use session, smokers were asked to use the same device used by the experienced EC users, *ad lib* for 10 min; before starting the session, a researcher explained to them the way EC are used, focusing on the coordination between activation of the EC device (by pressing the button) and puff initiation, and on the need for continuously pressing the button during the whole duration of the puff. 

All videos were uploaded to a personal computer and processed by video-processing software. Timing measurements were performed by frame-to-frame analysis. Videos were recorded at a frame rate of 25 s^−1^, thus the time between two consecutive frames was 40 ms. Puff, inhalation and exhalation duration was measured in both groups. For tobacco cigarettes, puff duration was the time interval between the frame at which the mouth was closed (with the cigarette filter-tip inside the mouth) until the frame at which the cigarette was removed from the mouth [13]. The interval from that frame until the frame just before visible smoke was exhaled was defined as inhalation time. Exhalation time was defined as the interval from the frame that smoke first became visible until the frame at which no more smoke was coming out of the mouth. For EC use, puff duration was defined as the interval between the frame when LED light was activated (with the mouthpiece inside the mouth) until the frame when the EC was removed from the mouth. Some users were activating the device before placing it in their mouths, while others did the opposite. We used the frame at which both LED was lit and mouth was closed as the timing of beginning of puff. EC inhalation and exhalation times were measured similarly to the measurements of the respective smoking times. Although participants may inhale from the nose during puff drawing, measurements of inhalation time in this study correspond to the inhalation of smoke and vapour from the oral cavity to the respiratory tract.

From every smoker, 10 consecutive puffs of smoking and 10 consecutive puffs of EC use were analysed. Similarly, 10 consecutive puffs of EC use from each experienced user of the device were also analysed, to equate to smoking one tobacco cigarette. For both groups, puffs 1–3 were not recorded, in order to allow users to get acquainted with the device; subsequently, puffs 4–13 were recorded and were used in the analyses. In order to reduce bias, different researchers examined the recordings of each study group. They were blinded to the results of other groups.

### 2.3. Evaluation of EC Liquid Consumption

Evaluation of EC liquid consumption was performed in experienced EC users only, during the same session when video recordings were made. The atomizer, filled with liquid, was weighed by a precision scale before use (minute 0), and after 5 and 20 min of use. The procedure of removing, weighing and repositioning the atomizer lasted approximately 25 s. The difference in weight between minute 0 and 5 was defined as the EC liquid consumption after 5 min of use (EC-C_5_). We used this five minute period because this is the approximate time needed for smokers to smoke one cigarette *ad libitum* [14]. This has been previously used in a study comparing the acute effects of EC with those of tobacco cigarettes [7]; therefore, the EC liquid consumption during that time was considered to be comparable to smoking one cigarette. The difference in weight between minute 0 and 20 was defined as EC liquid consumption after 20 min of use (EC-C_20_). The 20-min period was chosen because it represents the time needed to deliver 4 mg of nicotine from one cartridge of a nicotine inhaler [15]. The number of puffs was also recorded during the five and 20 minute periods for each participant.

### 2.4. Statistical Analysis

Continuous variables were expressed as mean ± SD and categorical variables as number (percentage). Intra-group comparisons in smokers (between smoking timings and EC use timings) were performed by using paired Student’s *t*-test. Intergroup comparisons were performed by using independent sample *t*-test for continuous variables and χ^2^ for categorical variables. Correlations were determined by using Pearson’s correlation coefficient. All comparisons were two-tailed, and a *p* value of <0.05 was considered statistically significant. To check for reproducibility of timing measurements, the intraobserver and interobserver mean error (the absolute difference between 2 measurements divided by the mean of the measurements) were calculated in 10 randomly selected studies in each group. Commercially available software was used for performing the statistical analysis (SPSS v18, Chicago, IL, USA).

## 3. Results and Discussion

### 3.1. Characteristics of Study Groups

The baseline characteristics of the study population are shown in Table 1. Both groups had similar age and gender distribution. Brinkman index (the product of daily number of cigarettes consumed and years of smoking) was higher in EC users due to slightly higher daily cigarette consumption and smoking duration.

**Table 1:** Baseline characteristics of the study groups.

Characteristic	Electronic cigarette users (n = 45)	Smokers (n = 35)	*p*-value
Age (years)	38 ± 5	37 ± 5	0.219
Gender (male)	35 (77.8%)	26 (74.3%)	0.716
Cigarette consumption (number per day)	26 ± 8	24 ± 5	0.125
Smoking duration (years)	21 ± 6	19 ± 5	0.151
Brinkman index	571 ± 258	451 ± 178	0.021
Electronic cigarette use (months)	7 ± 3		
Electronic cigarette consumption (mL/day)	5 ± 2		

Data expressed as mean ± SD or number (%). Brinkman index: product of daily number of cigarettes consumed and years of smoking.

### 3.2. Topography in EC Users Compared with Smokers

Significant differences were observed between ECIG and SM-S in vaping and smoking patterns (Table 2). Specifically, puff duration was double in ECIG compared to SM-S (*p* < 0.001). Similar difference was observed between ECIG and SME (*p* < 0.001). On the contrary, inhalation time was significantly lower in ECIG compared to both SM-S and SM-E. No difference was observed in exhalation time. Comparing smokers subgroups, SM-E had slightly higher puff duration (*p* = 0.017) while no significant differences were observed in inhalation and exhalation durations. The intraobserver and interobserver mean error values for each measurement in every group are shown in Table 1.

**Table 2:** EC and smoking pattern in the study groups.

Measurements	ECIG	SM-S	SM-E	*p*-value (ECIG *vs.* SM-S)	*p*-value (ECIG *vs.* SM-E)	*p*-value (SM-S *vs.* SM-E)
Puff duration (s)	4.2 ± 0.7	2.1 ± 0.4	2.4 ± 0.5	<0.001	<0.001	0.017
Inhalation duration (s)	1.3 ± 0.4	2.2 ± 0.4	2.0 ± 0.4	<0.001	<0.001	<0.001
Exhalation duration (s)	1.7 ± 0.5	1.8 ± 0.4	1.7 ± 0.3	0.379	0.614	0.426
Intraobserver error puff duration (%)	3.6 ± 0.6	4.2 ± 1.2	3.3 ± 1.2			
Interobserver error puff duration (%)	3.9 ± 1.4	5.2 ± 2.2	4.0 ± 1.6			
Intraobserver error inhalation duration (%)	5.8 ± 2.5	4.6 ± 1.2	4.9 ± 1.8			
Interobserver error inhalation duration (%)	8.3 ± 2.1	5.7 ± 1.5	7.0 ± 1.9			
Intraobserver error exhalation duration (%)	5.8 ± 2.6	5.0 ± 1.7	6.0 ± 2.0			
Interobserver error exhalation duration (%)	7.4 ± 1.5	6.6 ± 1.5	6.4 ± 2.1			

Abbreviations: ECIG: electronic cigarette users group; SM-S: smokers smoking tobacco cigarettes; SM-E: smokers using the electronic cigarette device. Data presented as mean ± SD.

### 3.3. Evaluation of EC Liquid Consumption

**Figure 2:**
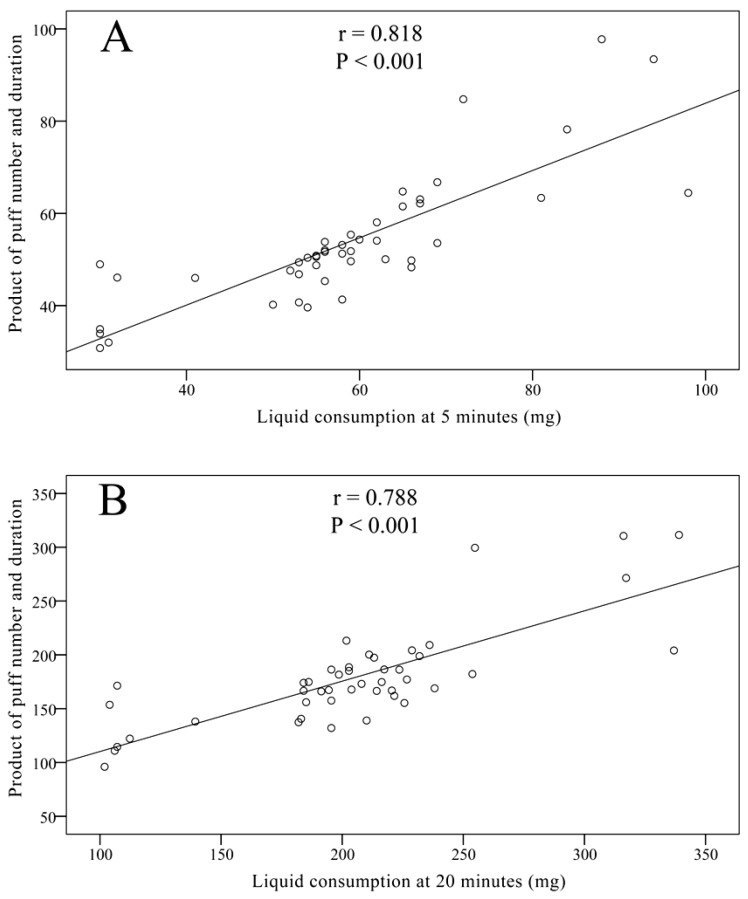
Correlation between electronic cigarette liquid consumption and product of puff number and duration, at (**A**) 5 and (**B**) 20 min of use.

EC users took 13 ± 2 puffs at 5 min and 43 ± 8 puffs at 20 min. EC-C_5_ was measured at 62 ± 16 mg while EC-C_20_ was 219 ± 56 mg. Significant correlations between puff number and consumption and between puff duration and consumption were observed for both 5 min use (r = 0.657 and r = 0.571 respectively, *p* < 0.001) and 20 min use (r = 0.663 and r = 0.523 respectively, *p* < 0.001). As expected, both EC-C_5_ and EC-C_20_ strongly correlated with the product of puff number and duration (Figure 2). No correlation was observed between consumption and age, EC duration of use, or smoking history.

**Figure 3:**
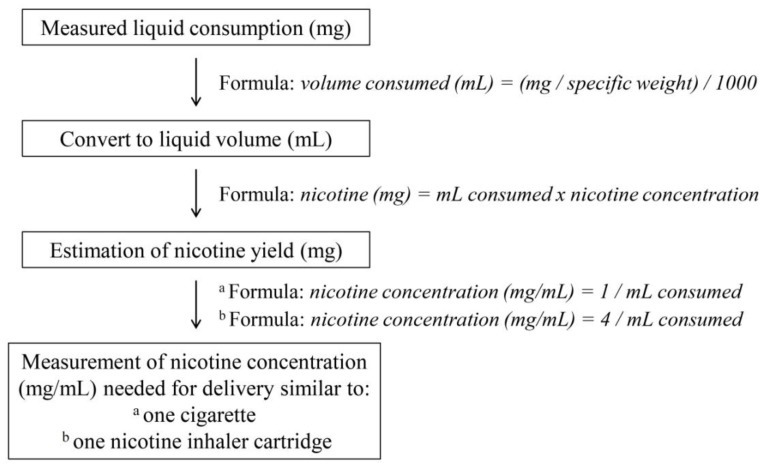
Schematic of the methodology used to estimate nicotine concentration of EC liquid needed to deliver nicotine amount similar to one tobacco cigarette and one nicotine inhaler cartridge.

The labelling of the EC liquid bottle reported nicotine concentration in weight per volume (mg/mL). In order to measure the amount of nicotine yield, weight measurements had to be converted to volume measurements. The formula used was: *volume consumed* (mL) *=* (mg*/specific weight*)/1,000. The specific weight of the liquid used was 1.21 g/mL. Therefore, weight measurements of liquid consumption corresponded to volumes of 0.052 ± 0.013 mL consumed in 5 min and 0.181 ± 0.046 mL consumed in 20 min. To measure the amount of nicotine delivered according to volume consumption, we used the formula: *nicotine* (mg) *=* mL *consumed* × *nicotine concentration*. Therefore, it was determined that, for the population studied, 5 min of EC use led to delivery of 0.46 ± 0.12 mg nicotine, while 20 min of EC use delivered 1.63 ± 0.41 mg nicotine. To estimate the nicotine concentration that would provide 1 mg of nicotine in 5 min (which is the highest allowable content in smoke from one cigarette measured by the ISO method [16]) based on the measured consumption, we used the formula: *nicotine concentration* (mg/mL) *=* 1*/*mL *consumed.* It was estimated that, in order to deliver 1 mg nicotine to our study sample, liquid with 20.8 ± 6.3 mg/mL nicotine concentration should be used. For estimating nicotine concentration that would deliver 4 mg nicotine in 20 min, we used the formula: *nicotine concentration* (mg/mL) *=* 4*/*mL *consumed*. By this formula, it was found that 23.8 ± 7.2 mg/mL nicotine-concentration liquid should be used. The methodology used for these measurements is presented in Figure 3.

### 3.4. Discussion

This is the first study specifically designed to assess EC use (vaping) topography in experienced users. The difference between cigarette smoking and EC use topography was also examined, and a direct comparison of EC use was made between experienced and novice users. The results of this study show that there is a significant difference between vaping and smoking topography, with experienced EC users taking longer puffs. The use of video recordings allowed us to measure inhalation time, which was significantly lower in experienced vapers when using the EC compared to smokers when smoking. A difference was also observed when comparing EC use between experienced and novice users, confirming previous assumptions that experienced vapers use the device more intensively [5,8]. Finally, by assessing EC liquid consumption in experienced users at various time intervals, a measure of nicotine delivery was provided and comparisons could be made with tobacco cigarettes and NRTs.

#### 3.4.1. Vaping *vs.* Smoking Topography

Tobacco cigarette topography has significant implications on the amount of nicotine and other chemicals delivered to the smoker [17]. Traditionally, the ISO smoking method (35 mL puffs in 2 s, 60 s interpuff interval) has been used, although it seems to underestimate the intensity of real use in terms of puff volume and interpuff interval [18,19]. ECs have a unique mode of function; therefore, vaping topography was expected to be different. Unlike tobacco cigarettes, which are continuously burned at similar temperatures during the whole time of use, ECs undergo repeated thermal cycles of heating and cooling. Initially, the device is at room temperature and the resistance and wick are impregnated with liquid. When it is activated, heat is produced until the boiling point of the liquid is reached; then the energy is used to transform liquid into vapour. After completing the puff, no energy is delivered to the resistance and wick, so the temperature is progressively decreasing; at the same time, liquid is re-supplied to the wick encircled by the resistance, further decreasing its temperature. This study showed that puff duration during vaping is almost double compared to smoking. This might be partly attributed to a delay from the time of activation until the beginning of evaporation and to lower rate of vapour production from the EC device compared to smoke production when smoking a tobacco cigarette. On the contrary, inhalation time was lower in EC use. This could be attributed to the fact that higher inhalation time significantly decreases the amount of vapour exhaled. A phenomenon of “stealthy vaping” is described by EC users, wherein users hold their breath for a few seconds after vapour inhalation which results in no visible vapour being exhaled. Since the presence of visible vapour resembles smoke exhalation from smoking tobacco cigarettes, it can be assumed that vapers lower inhalation time in order to see more vapour exhaled and therefore reproduce one of the rituals of smoking [20]. Smokers who were using the EC device for the first time took shorter puffs, confirming previous observations that there seems to be a learning curve in the use of ECs [5]. Therefore, in order to realistically examine the effects of EC use in clinical studies, either experienced users should be recruited or naïve users (smokers) should be asked to double their usual puff duration and reduce their inhalation time.

#### 3.4.2. Implications from Evaluating Vaping Topography

Vaping topography may have significant implications in production and delivery of potentially harmful substances. The EC evaporation rate and thermal load are directly dependent on the puff duration and interpuff interval. If the device is activated before the temperature is significantly decreased and/or before the wick is sufficiently supplied with liquid, the device will get overheated. This causes a phenomenon called “dry puff”. It is an unpleasant, burning taste that forces the user to lower puff duration and increase interpuff interval. It is also reproduced when the atomizer has very low amounts of liquid, signalling that it should be refilled. This phenomenon occurred in some experienced users when they were asked to use the “eGo-C” atomizer in this study. They had to lower puff duration and interpuff interval in order to avoid “dry puff”, while no such problems occurred with the “Epsilon” atomizer. Although not tested yet, there is a theoretical concern that overheating the EC may lead to production of significant amounts of toxic substances like acrolein or formaldehyde, which can be formed from thermal degradation of glycerol in a closed chamber [21,22]. The “dry-puff” phenomenon, although easily detected and avoided by the user, cannot be detected in the laboratory setting. Therefore, if this occurs during a laboratory experiment, it will significantly undermine the value of the study results and their applicability to real use. It should be emphasized that each type of atomizer has different cooling and liquid-supply abilities, depending on the design and material used. This should be taken into consideration when preparing laboratory research protocols. This study provides information on the way EC devices should be handled in laboratory experimental settings. Four-second puffs should be taken, with 20–30 s interpuff interval. Using ECs in a way similar to tobacco cigarettes (2 s puffs with 60 s interpuff interval) may underestimate the potential risks from pragmatic EC use. However, it should be emphasized that information on vaping topography from this study applies only to atomizers which can support such vaping patterns without reproducing the dry-puff phenomenon. Using the same puff duration and interpuff interval with less sufficient atomizers would mean that the experimental conditions are not applicable to real use and risks could be overestimated. In this study, a battery device which, according to major retailers, represents more than 85% of sales in the Greek EC market was used. A newer-generation atomiser was also used, which seems to work more efficiently compared to older designs and is very popular among users who have successfully reduced or completely substituted smoking with EC use. In any case, it is important that an experienced EC user tests the conditions of use in order to determine whether the “dry puff” phenomenon occurs, because there is no other method to detect it. 

#### 3.4.3. Nicotine Delivery

Comparative levels of nicotine delivery between electronic and tobacco cigarettes or NRTs had not been previously established. This is important from a public health perspective, especially in the context of the proposal for a new TPD by the European Commission [9] which in reality proposes that most of currently available liquids will be excluded from the market unless they are approved as medicinal products. Discussion about whether ECs should be considered medicinal products is beyond the scope of this study; however, the EC liquid consumption of experienced users during *ad lib* use was examined, at time-periods similar to smoking one cigarette (5 min) [7] and obtaining 4 mg of nicotine from a nicotine inhaler (20 min) [15]. Although a 9 mg/mL nicotine-containing liquid was used, nicotine delivery to EC users was still 54% lower compared to a 1 mg-nicotine tobacco cigarette and 59% lower compared to a nicotine inhaler. This provides a plausible explanation for the fact that the majority of EC users consume liquids with nicotine concentration much higher than 4 mg/mL, as reported in several internet forums and observed in surveys [12,23]. It was calculated that, in order to deliver nicotine amount similar to one nicotine-inhaler cartridge or one tobacco cigarette, liquids of 20–24 mg/mL nicotine concentration should be used. Therefore, the reasoning for selecting 4 mg/mL nicotine concentration as comparable to NRTs in the TPD cannot be supported by the results of this study. Low-nicotine liquids are probably ineffective in substituting smoking, especially during the initial period of use, and this has been confirmed by studies showing that most commonly-used EC liquids have 18 mg/mL nicotine concentration [12,23]. There is concern that reducing the availability of effective liquids might lead to increased cigarette consumption by current EC users (or even smoking relapse in those who have completely substituted smoking). This would compromise the position of ECs in the field of tobacco harm reduction, a strategy of significantly reducing harm by using them as an alternative-to-smoking habit [24,25]. It may also have significant health implications, since we know that smoking produces subclinical dysfunction even at young age [26], while reducing cigarette consumption can have beneficial effects on cardiovascular risk and lung cancer mortality [27,28,29]. A review of chemical analyses of EC liquid found that nitrosamines were present at levels 500–1,400 times lower compared to smoking [24]. Goniewicz *et al.* observed that the amount of carbonyls produced by EC use in laboratory conditions was 9–450 times lower compared to tobacco cigarettes [4]. Thus, it can be assumed that they are considerably less harmful compared to tobacco cigarettes. However, it is not known, and is still too early to evaluate, whether reduction or complete substitution of smoking by EC use has any long-term health benefits.

#### 3.4.4. Limitations

Some limitations certainly apply to this study. For the purpose of calculating nicotine delivery, the levels of nicotine present in liquid were compared with the levels present in cigarette smoke. Unburned tobacco cigarette contains significantly higher levels of nicotine compared to what is present in the smoke, since nicotine is heat-sensitive. Heat produced by the EC might also result in lower amount of nicotine being delivered in vapour form compared to the amount present in liquid, although temperature is far lower compared to tobacco combustion. The ISO-measured nicotine delivery of cigarette smoke (reported in cigarette packaging) was used as a comparison, which has been shown to underestimate true nicotine delivery from smoking by approximately two-fold [30]. Lower inhalation time was observed with EC use compared to smoking, and this might further decrease nicotine delivery to and absorption from the lungs, although this remains to be studied. Finally, EC users participated in the study after abstaining from EC use for at least eight hours; this probably resulted in more intense use of the device compared to daily normal use. All the above strengthen the results of this study and imply that, in order to deliver amounts of nicotine similar to a tobacco cigarette, liquids should probably have higher than 24 mg/mL nicotine concentration. However, nicotine absorption was not measured. It has been documented that nicotine is absorbed at a rapid rate from the lungs by tobacco cigarette use [31], while it is slowly absorbed from the buccal mucosa by nicotine inhaler use [15]. Currently we do not have sufficient data to determine the absorption pharmacokinetics of nicotine from EC use, and this should be examined. Moreover, there are no studies about the safety of long-term vapour inhalation from ECs.

Video recordings were used for evaluating vaping and smoking topography, instead of a computerized method (automated smoking topography machine). Blank *et al.* found that measurements differed little between the two methods and they were both considered reliable when evaluating smoking topography [13]. Moreover, by using video recordings inhalation time could be estimated, which was found to be lower in EC users compared to smokers. This could not have been measured by using a smoking topography machine. However, it is unknown how other confounders could affect vaping topography, like higher nicotine concentration liquids, different flavours and differences in liquid ingredients (different mixtures of propylene glycol and glycerol); future research should be focused on these issues. To avoid compensatory changes in topography due to nicotine levels, EC users who were daily consuming liquids with nicotine concentration similar to that tested we recruited and examined.

## 4. Conclusions

In conclusion, vaping topography was significantly different compared to smoking topography, indicative of the fundamental differences in the use patterns between the two products. Experienced users use the EC more intensively compared to novice users; therefore, clinical studies evaluating EC effects by recruiting novice users should be interpreted with caution. Finally, our findings suggest that nicotine delivery is significantly reduced by EC use compared to cigarette smoking and nicotine inhaler at similar use-periods, and this should be considered by the public health authorities for their regulatory plans.
